# Microsurgical Resection of a Diaphragma Sellae Meningioma via Extradural Clinoidectomy with Preservation of Superior Hypophyseal Arteries

**DOI:** 10.1055/a-2765-5582

**Published:** 2025-12-15

**Authors:** Umid Sulaimanov, Jiyang An, Ufuk Erginoglu, Cagdas Ataoglu, Yerkebulan Serikkanov, Abdullah Keles, Mustafa K. Baskaya

**Affiliations:** 1 Department of Neurological Surgery, School of Medicine and Public Health, University of Wisconsin-Madison, Madison, Wisconsin, United States

**Keywords:** anterior clinoidectomy, diaphragma sellae meningioma, microsurgical resection, optic nerve decompression, pterional approach, skull base surgery

## Abstract

Diaphragma sellae meningiomas are rare suprasellar tumors often misidentified as tuberculum sellae meningiomas. Their association with the optic nerve, chiasm, pituitary stalk, internal carotid, and superior hypophyseal arteries presents unique surgical challenges. These tumors are classified into three types based on dural attachment with precise subtype identification. We present a Type A diaphragma sella meningioma, located anterior to the pituitary stalk, managed via a pterional craniotomy with extradural anterior clinoidectomy and optic unroofing. Posterior attachment with ICA adhesion and multiple SHA involvement favored a transcranial route, with clinoidectomy and optic unroofing widening the optico-carotid triangle for safe resection.

## Transcript ( Video 1 ; mm:ss)

**Video 1.** Key microsurgical steps of diaphragma sellae meningioma resection performed via a pterional craniotomy with extradural anterior clinoidectomy and optic nerve unroofing, demonstrating preservation of the superior hypophyseal arteries.


## 0:00 Introduction

This video demonstrates the important steps for the microsurgical resection of a diaphragma sella meningioma using the pterional transsylvian approach.

## 0:07 Background Information

Anterior skull base meningiomas account for approximately 9% of all meningiomas. 1 Diaphragma sellae meningiomas are rare meningiomas arising from arachnoid cap cells within the dural fold forming the roof of the sella turcica, which contains a central opening for the pituitary stalk. These lesions typically grow in the suprasellar or intrasellar space, displacing the optic apparatus and pituitary gland. 2 Historically grouped with tuberculum sellae meningiomas as “suprasellar meningiomas,” these were first clearly distinguished by Busch and Mahneke in 1954. 3 This was then extended by Kinjo et al who classified these based on their dural attachment as: Type A, anterior to the stalk and often causing unilateral visual loss; Type B, posterior to the stalk and frequently producing hypopituitarism; and Type C, intrasellar, where are associated with bitemporal hemianopsia and endocrine dysfunction. 2

## 0:58 Case Presentation

We demonstrate here the key steps for microsurgical resection of a Type A diaphragma sella meningioma via a right pterional transsylvian approach.

The patient is a 60-year-old woman with a slowly growing diaphragma sella tumor, first diagnosed approximately 10 years ago at another medical center. The patient was referred to us for further evaluation and surgical consideration after noting a slight enlargement compared to prior MRIs. Ophthalmological examination at our institution was unremarkable. Brain MRI demonstrated a suprasellar mass which seemed to arise from the diaphragma sellae. Given the tumor's size, location, relatively young age of the patient, and slight growth over time, surgical intervention was advised.

## 1:37 Preoperative Imaging

Brain MRI demonstrated a well-circumscribed, homogeneously contrast-enhancing suprasellar mass measuring approximately 1.5 × 1.6 cm, that displaced the infundibulum posteriorly and the optic chiasm superiorly. We thus diagnosed this as a diaphragma sellae meningioma.

## 1:55 Management

Surgical approach selection is guided by tumor size, extension to the contralateral side, and, in particular, vascular involvement. Surgical options include transcranial or endoscopic endonasal routes, with each offering distinct advantages and disadvantages for effective resection. 4 5 6 7 The endonasal approach is generally favored for midline tumors with limited lateral extension and no significant vascular involvement, as it permits early tumor devascularization and direct access without brain retraction. Conversely, transcranial routes remain the most established approach and provide better access to tumors extending laterally beyond the optic nerves or those encasing arteries, allowing for safer dissection under direct visualization. 8 9 In select complex cases, a tailored or combined approach may be required to maximize resection while minimizing neurological morbidity. In this case, posterior attachment with ICA adhesion and multiple SHA involvement, including branches to the pituitary stalk, favored a transcranial route. By adding clinoidectomy, optic canal unroofing, and sectioning the falciform ligament, the optico-carotid triangle was widened to permit safe handling of this vasculature.

## 2:42 Patient Positioning and Skin Incision

In the present case, given the size and location of the diaphragma sellae meningioma, a transcranial approach with extradural anterior clinoidectomy and optic unroofing was performed to provide wide exposure of the tumor and surrounding neurovascular structures, particularly the optic nerve, superior hypophyseal arteries, and pituitary stalk. This afforded safe and effective dissection, enabling maximal tumor removal with preservation of critical anatomy and minimizing the risk of complications, with the primary goal of achieving gross total resection while ensuring patient safety.

Under general anesthesia, the patient was positioned supine, with her head turned to the left and held with three-point fixation. A curvilinear incision behind the hairline was marked, followed by a standard pterional craniotomy. The lesser sphenoid wing was drilled, and the meningo-orbital band was coagulated and divided. At this point, we prefer to introduce the operating microscope to begin early extradural dissection, anterior clinoidectomy, and optic nerve unroofing. After sectioning of the meningo-orbital band, we continued with elevation of the dura off the anterior skull base and optic roof, as well as off the lateral wall of the cavernous sinus. This step makes the field shallower and wider for safer clinoidectomy. The superior orbital fissure, V1, and V2 were all exposed next. We then placed stay sutures to retract the dura. We find that this dural suture retraction provides a much lower profile than self-retaining retractors, which may also occupy the field, and that this protects the brain from retraction forces. We also find that clinoidectomy and optic unroofing increase the exposure of the optico-carotid triangle and widen the working angle infero-laterally to the ipsilateral optic nerve. This also allows incision of the falciform ligament at the intradural surgical stage, which prevents microtrauma during manipulation of the optic nerve, which is strangulated by the ligament due to compression from the meningioma. 10 And finally, this overall approach facilitates broader exposure of tumor components extending into the optic canal.

## 4:34 Dural Opening and Arachnoid Dissection

The dura was opened in a curvilinear fashion, pedicled anteriorly, and reflected. The arachnoid of the proximal limb of the Sylvian fissure was sharply opened, exposing the M1 segment of the middle cerebral artery, and traced back to the ICA bifurcation. The ICA was identified, and the optico-carotid cistern was opened, revealing the tumor within it. The ipsilateral A1 segment was dissected to the anterior communicating complex, elevating the frontal lobe off the optic nerve. Multiple fenestrations of the A1 and anterior communicating complex were noted bilaterally, with accessory A1 arteries present on both sides.

The tumor was also present within the prechiasmatic cistern. One of the superior hypophyseal arteries was identified on the tumor surface lateral to the ipsilateral optic nerve within the optico-carotid triangle. This artery was carefully dissected free and preserved. We recommend that this microvascular dissection be done before entering the tumor.

The falciform ligament was sharply divided to allow mobilization of the optic nerve, which was carefully freed from the tumor surface. This maneuver enhanced the benefit of the prior clinoidectomy by further decompressing the optic nerve, relieving tethering, and facilitating its gentle, trauma-free mobilization while minimizing the risk of microtrauma. Together, the clinoidectomy and falciform ligament opening provided wide exposure while significantly facilitating access to the tumor base.

The tumor was then entered both anterior and posterior to the optic nerve. It was predominantly soft, purple, and friable. The capsule was coagulated with bipolar cautery to reduce its bulk, and the tumor was meticulously microdissected away from both optic nerves, the remainder of the optic apparatus, and the ipsilateral carotid artery. Multiple superior hypophyseal arteries were identified in the operative field, necessitating meticulous dissection to preserve their integrity and prevent infarction or microinfarcts in the pituitary gland and optic chiasm. Finally, the tumor was devascularized by bipolar coagulation along its dural base.

## 6:42 Tumor Resection

The tumor was internally debulked, with representative samples sent for permanent pathology. Throughout this stage, tumor debulking was alternated with microsurgical dissection to reduce tumor volume and progressively enhance visualization. An ultrasonic aspirator was used in parallel with sharp dissection to enable circumferential mobilization of the tumor and facilitate atraumatic dissection around critical structures. We recommend the use of low-intensity ultrasonic aspiration to avoid laceration of the vasculature. Following debulking, the ipsilateral portion of the tumor was carefully dissected free from all its attachments within the optico-carotid triangle.

Once this was completed, attention was turned to the anterior aspect of the tumor, which was then dissected from the contralateral optic nerve and the contralateral superior hypophyseal artery.

The tumor was further separated from all remaining critical structures, including the diaphragma sellae and from the pituitary stalk, along with another superior hypophyseal artery that was preserved. Once fully detached, the residual tumor was removed and also sent off to pathology. At this stage, satisfactory resection had been achieved. Topical nitroprusside solution was then applied to the vessels and optic apparatus to prevent the consequences of mechanical vasospasm. Intradural hemostasis was achieved. The dura was closed in a watertight fashion, the bone flap was secured using a plating system, and the wound was closed in anatomical layers.

## 8:31 Postoperative Imaging and Course

The surgery and postoperative course were uneventful, with the patient then awakening neurologically intact and unchanged from the baseline. Postoperative MRI revealed gross-total resection. Histopathological analysis confirmed a WHO grade I meningioma. Follow-up MRI at 3 months demonstrated no evidence of residual or recurrent tumor.

## 8:49 Conclusion

Both transcranial and endoscopic endonasal approaches can be effective for managing diaphragma sellae meningiomas, though microsurgical transcranial approaches remain the most established and versatile, particularly for complex anatomy. This approach provides excellent exposure and allows careful dissection of the tumor away from critical structures, including the optic nerves, carotid arteries and their branches, the pituitary stalk, and especially the superior hypophyseal arteries. Early extradural anterior clinoidectomy and meticulous arachnoid dissection help create a safe working corridor while facilitating maximal resection and minimizing risk. In the event of vascular complications, having open access allows for rapid and controlled management of vascular injury. Careful preoperative planning and a stepwise microsurgical technique remain important for safe tumor removal, highlighting the utility of transcranial microsurgery for complex anterior skull base cases.
